# The Development of Alcoholic Subtypes: Risk Variation Among Alcoholic Families During the Early Childhood Years

**Published:** 1996

**Authors:** Robert A. Zucker, Deborah A. Ellis, C. Raymond Bingham, Hiram E. Fitzgerald

**Affiliations:** Robert A. Zucker, Ph.D., is professor of psychology in the Departments of Psychiatry and Psychology at the University of Michigan and director of the University of Michigan Alcohol Research Center, Ann Arbor, Michigan. Deborah A. Ellis, Ph.D., is an assistant professor in the Department of Psychiatry and Behavioral Neurosciences, Wayne State University School of Medicine, Detroit, Michigan. C. Raymond Bingham, Ph.D., is a research assistant professor in the Department of Psychology, Michigan State University, East Lansing, Michigan. Hiram E. Fitzgerald, Ph.D., is professor and associate chairperson in the Department of Psychology and coordinator of the interdepartmental graduate specialization in applied developmental science at Michigan State University, East Lansing, Michigan

**Keywords:** disorder classification, AOD dependence, risk assessment, risk factors, high risk group, familial alcoholism, children of alcoholics, childhood, child, family environment, family dynamics, antisocial behavior, hyperactive behavior, aggressive behavior, behavioral problem, adolescence, longitudinal study, prospective study

## Abstract

Lifetime differences in antisocial behavior among alcoholic men historically have been useful in distinguishing alcoholic subtypes. However, the usefulness of this subtyping strategy for identifying differences in families that may put offspring at risk for developing later alcoholism has not been previously documented. Findings from a prospective study on the development of vulnerability for alcoholism among (initially) preschool-age children showed that children from families with antisocial alcoholism differ on a number of indicators of child risk, including measures of risky temperament, externalizing behavior problems, and hyperactivity. Risk differences among children from these family subtypes appear to be sustained into middle childhood. Differences between nonantisocial alcoholic families and nonalcoholic control families were less distinguishable in both early and middle childhood.

The diagnostic system most widely used for classifying psychiatric disorders is the *Diagnostic and Statistical Manual of Mental Disorders* (i.e., the DSM criteria). The most recent version of this manual, the fourth edition (American Psychiatric Association 1994), provides two categories in which to classify alcohol-use disorders: (1) alcohol dependence, which generally involves symptoms of tolerance and withdrawal, along with a host of other symptoms indicative of chronic and compulsive alcohol use, and (2) alcohol abuse, which involves symptoms that are less chronic and severe. Instead, the critical identifying feature for alcohol abuse is a pattern of use characterized by recurrent and significant adverse consequences. Close examination of the DSM criteria shows that even within these two categories, a multiplicity of different symptom complexes and outcomes exists. Both researchers and clinicians have been aware of the diversity of alcohol abuse and alcoholism (i.e., its heterogeneity) for well over a century (Babor and Lauerman 1986), and the DSM–IV classification system is the most recent, albeit imperfect, attempt to sort these varied symptoms in a meaningful way.

Alcohol abuse involves a complex of behaviors. These behavior characteristics are less severe and they change rapidly (i.e., are more transitory) compared with the characteristics of alcohol dependence. As a result, the use of two distinct categories for classifying people with alcohol problems seems justified. In addition, as noted in the DSM–IV, abuse does not invariably lead to dependence. Moreover, signs of early alcohol dependence do not necessarily indicate that an individual will continue this pattern of behavior over time (Skog and Duckert 1993). Because such heterogeneity exists within this classification system, scientists continue to speculate that the causes underlying the apparently single disorder of alcohol abuse-dependence may actually involve multiple processes (Cloninger 1987; Cloninger et al. 1986; Hesselbrock 1995; Schuckit 1985; Zucker 1994). Scientists continue to look for other categorization approaches that would better reflect the variability that exists within the alcohol abuse-dependence framework. This is the rationale behind the search for subtypes.

One study that has documented the heterogeneity found within alcohol abuse and dependence is the Epidemiologic Catchment Area (ECA) Study. This study provided a survey of the distribution of psychiatric disorders. Using these data, scientists have been able to project the prevalence of alcohol abuse-dependence within the U.S. population as well as begin to make estimates of the extent to which this disorder is associated (i.e., the degree of aggregation) with other psychiatric syndromes, such as antisocial personality disorder (ASPD) (Helzer and Pryzbeck 1988; Regier et al. 1990). The ECA study has documented that significant variation exists in the degree of aggregation of alcohol abuse-dependence with other psychiatric disorders. For example, although ASPD occurs in only 4 percent of the U.S. male noninstitutional population, it is 12 times more common among those with alcohol abuse-dependence than it is among those without the alcohol-use disorder. Less dramatic, but also suggestive of aggregation, is the association, particularly in women, between mania and alcohol abuse-dependence. Mania occurs in less than 1 percent of the general population of women; however, the chances of depression being present are nine times greater among woman with alcohol abuse-dependence. These aforementioned associations may be possible indicators of different alcohol-disorder subtypes (Babor and Dolinsky 1988; Zucker 1994). If this is the case, the comorbid psychiatric disorders would help in identifying alcoholism^1^ subtypes that are clinically more alike (i.e., homogeneous).

Several possible theories exist to explain how alcohol abuse-dependence may be linked to other psychiatric disorders. One hypothesis states that because alcohol abuse-dependence and other disorders occur together, these disorders must share a common developmental process (i.e., etiology). An alternative possibility, and one that must be ruled out to fully understand the co-occurrence of these disorders, is that the psychiatric symptoms are simply a result of the alcoholism, rather than a marker of a common causal process. Still another possibility is that the symptoms of alcoholism and other psychiatric disorders occur independently but share a common factor that contributes to the development of both disorders.^2^ By determining how alcoholism relates to psychiatric symptoms, researchers may be able to determine which explanation is the most likely. Researchers would then come closer to identifying the subtypes that best reflect the heterogeneity of the disorder. Such specific descriptions would be useful as indicators of potential differences in the course and causes of the disorder and could possibly assist in the development of prevention and treatment strategies.

**Figure:**
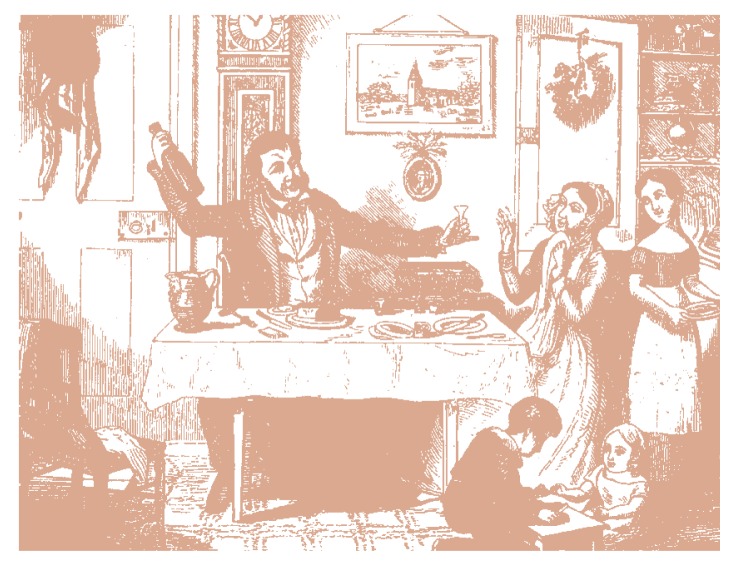
Alcoholism risk factors illustrated in “The Bottle,” 1847, by George Cruikshank. Reproduced with permission from the *Journal of Studies on Alcohol*. © Alcohol Research Documentation, Inc., Rutgers University Center of Alcohol Studies.

In both men and women, alcoholism is associated most strongly with the comorbid disorder ASPD. This disorder is characterized by a pervasive disregard for and violation of the rights of others and is evident during both childhood and adulthood. The presence or absence of symptoms composing ASPD is a major distinguishing feature of virtually all of the alcoholism subtyping schemes developed during the past generation (Babor and Dolinsky 1988; Cloninger et al. 1981; Hesselbrock et al. 1984; Babor et al. 1992; Zucker 1994; Zucker et al. 1994). Although it is less commonly acknowledged, children from families with alcoholic adults who have antisocial symptoms (i.e., who have high levels of antisocial symptomatology) are at greater risk of becoming alcoholic later in life than other children. (For a definition of risk, see sidebar.) The risk factors include having a greater number of alcoholic relatives (i.e., a denser family history of alcoholism), which, in turn, will increase the probability that the children will have some genetically mediated vulnerability for alcoholism; more severe alcoholic symptoms and more nonalcoholic psychiatric symptoms among the parents; and a greater likelihood that a variety of relational problems (e.g., marital and legal problems) exist within the family (Hesselbrock et al. 1984; Lewis 1990).

**A Note About Life-Course Variation and the Development of Risk**

The concept of risk refers to the statistical probability that a specific (usually negative) outcome will occur at a later date (Zucker 1989). Thus, when identifying risk characteristics for the later emergence of alcoholism among children, researchers understand that they are making a similar probability statement that—all other things being equal—a given pattern of influences will increase or decrease the probability of a later harmful outcome. The phrase “a pattern of influences” implies that a number of factors are present and that they must operate in concert, rather than in opposition, for the disorder to ultimately appear. Consequently, in families with alcoholism in which antisocial characteristics also are high, potential influences found in early childhood might include a heightened genetic vulnerability for alcoholism, a temperament that generates problematic responses from others, a rearing environment that may encourage problem alcohol use, and a family structure with conflict within its boundaries. Such influences, in turn, are related to the presence or absence of other psychiatric symptoms in one or both of the parents. The phrase “all other things being equal” refers to the fact that time passes, and as it does, other influences appear that also may affect outcome. As the child grows older, school provides another rearing environment, as do peer relationships outside the family. Social conditions do not always exacerbate problematic temperamental styles; they sometimes operate to dampen such behavior (Forehand and McMahon 1981). Only when these factors operate together are the outcomes likely to be of the highest risk, the greatest damage, and the earliest appearance of difficulty.

***High-Risk Longitudinal Studies***

Tracking and understanding how risk factors operate together is a significant challenge. The influences (i.e., the causal structures) that must be assessed are not available all at once. In fact, these influences are likely to emerge gradually at different points during the life course. The impact of each influence also may be seen only gradually, as patterns of behavior become shaped and consolidated.

The research method of choice for mapping the structure of such influences is the longitudinal study. To highlight specific processes, investigators use the high-risk longitudinal study. In such studies, individuals are selected who are known to differ in their likelihood of later showing signs of the disorder—in this case, alcoholism. The causes for the ultimate outcome are not known; the selection of risk groups is based on the statistical likelihood of developing the disorder, rather than on an understanding of how the risk status is manifested. Statistically speaking, outcome is predictable, only at the study group level. By choosing a network of variables that, optimally, includes those factors which are the ultimate causes for the disorder and by tracking study participants over time, researchers are able to document earlier characteristics that influence the later disorder, characteristics that might be protective and insulating against it and characteristics that ultimately are irrelevant to later clinical outcome.

—*Robert Zucker*

ReferencesForehandRMcMahonRHelping the Non-compliant Child: A Clinician’s Guide to Effective ParentingNew YorkGuilford Press1981ZuckerRAIs risk for alcoholism predictable? A probabilistic approach to a developmental problemDrugs and Society469931989

The variations in antisocial symptoms found in adult alcoholics offer a potentially powerful framework (i.e., construct) on which to base future subtypes. A collaborative group of researchers from three Michigan universities have been working to further evaluate and refine this construct. The goal of this research effort is to better define the variations in symptoms found in individual adult alcoholics and to delineate the differences that exist among the families (i.e., familial variations). The hypothesis guiding this research is that these familial variations will influence the likelihood that the children from these families will develop alcohol problems or alcoholism later in their lives.

## The Michigan State University-University of Michigan Longitudinal Study

The Michigan State University-University of Michigan (MSU–UM) Longitudinal Study (Zucker 1987; Fitzgerald et al. 1995) began as a pilot study in 1982, and researchers began regular data collection in 1985. The MSU–UM study was set up according to a high-risk design structure (see sidebar), and it is tracking high-risk families that include a heterogeneous group of 220 alcoholic men, their initially preschool-age (i.e., 3 to 5 years old) sons, and the boys’ biological mothers. The plan is to continue the study well into the children’s adulthood. When the study began, the mothers’ drinking status ranged from alcoholic to nondrinker. Families were excluded from participating, however, if the child displayed signs of fetal alcohol effects. Mothers and fathers had to be living together at the beginning of the study; however, as is common in alcoholic families, separation and divorce occurred at high rates. Even in such cases, the study continues to follow both biological parents. If a custodial parent has remarried, the custodial stepparent is added to the study. In addition to this high-risk group, the study includes a contrast group of 91 families with similar structures located in the same neighborhoods as the high-risk families; however, in these families, both parents were free of alcoholism and other drug (AOD) dependence.

Boys initially were selected as the target group because in the general population alcoholism is approximately five times more common in men than in women. In addition, sons of alcoholic fathers are about 1.5 times more likely to develop alcoholism than the offspring of nonalcoholics (Russell 1990). A parallel study tracking the risk for alcoholism among girls would require a much greater number of subjects and, consequently, a much more expensive design. Nonetheless, the outcome for girls from alcoholic homes is an equally important area of investigation, given the broad range of other difficulties that female children of alcoholics (COA’s) experience (Cloninger et al. 1986; Goodwin et al. 1977; Widom 1993). An addition to the study has allowed the project to include one daughter from each of the families studied in cases where this option is available. It is still too early, however, to evaluate the data obtained from these girls.

Families are assessed at 3-year intervals. Although the study has continued for more than a decade, all parents but one continue to participate, including those who have moved away from the study’s primary field site.^3^ At each time point, or wave, of data collection, family members participate in a nine-session schedule in which an extensive set of measures is used. These measures include interviews; self-report questionnaires; reports by collateral informants, such as spouses, parents, and teachers, as well as the children’s reports of their experiences with their parents; observer ratings; and data obtained from videotaped interactions. Some research is conducted at the university laboratory, but most data are collected in the respondents’ homes to ensure cooperation from a study population that is known for its waywardness and chaos. Data collectors do not know the families’ risk status.

## Early Results From the MSU–UM Longitudinal Study

Although the study’s ultimate outcome can be determined only after the children reach adulthood, a number of influencing structures are likely to play a role in shaping the development of alcoholism. These influences include differences in genetic vulnerability;^4^ rearing environment variations; cultural, community, and socioeconomic influences associated with risk for alcoholism (i.e., macrolevel environmental factors); and, most important, the child’s personal characteristics that may put him or her at risk for an alcoholic outcome. To assess these influences, the study uses a number of measures that are proxy indicators of risk load. So far, the indicators being used are measures of externalizing behaviors (e.g., aggression, hyperactivity, and delinquency) because these characteristics are known to be precursors to antisocial deviance, which, in turn, has repeatedly been shown to be a precursor to the development of AOD abuse in adolescence (see Kandel 1978).

### Early Risk Variation Among the Families

Using data from the MSU–UM study’s initial assessment period (i.e., when the children were ages 3 to 5), a series of analyses evaluated differences in the home rearing environments and in the presence and extent of externalizing behavior in the children from the alcoholic (i.e., high-risk) versus the nonalcoholic (i.e., low-risk) families. These analyses demonstrated a number of significant differences between the high- and low-risk groups, as follows:

- Alcoholic parents exhibited greater levels of psychopathology (e.g., depression and antisocial symptomatology) than nonalcoholic parents.
- The quality of the home rearing environment, as assessed by an interview and observation measure of the cognitive, social, and emotional stimulation available to the child, was poorer in the high-risk than in the low-risk families (Fitzgerald et al. 1993; Noll et al. 1992; Whipple et al. 1995).
- Although both groups were recruited from the same neighborhoods, high-risk families were lower on indices of social functioning and access to societal opportunities than were the low-risk families (i.e., the high-risk parents were of a lower socioeconomic status and had less education) (Fitzgerald and Zucker 1995).
- COA’s demonstrated higher levels of externalizing behavior than non-COA’s and were more likely to exhibit the difficult temperament characteristics (e.g., high activity level) that Tarter and colleagues (1995) hypothesized were precursors to later alcoholic outcome (Jansen et al. 1995).
- Although they were still preschoolers, the COA’s could more readily identify alcoholic beverages. They also were more likely to expect male adults to choose alcoholic drinks as the beverages of choice in everyday social situations. These findings show that the COA’s have a more developed cognitive structure concerning alcoholic beverages. Thus, despite their young age, the two groups of children already differed in their rudimentary alcohol expectancy structure (Zucker et al. 1995*a*).

## Subtyping of Alcoholic Families

As previously described, significant differences were found between the high- and low-risk families. The study’s interest in identifying different patterns of risk variation led the investigators to explore whether risk aggregation might be even more concentrated if the parents’ alcoholism were subtyped. The subtyping scheme used was a classification based on the presence or absence of differences in each father’s antisocial behavior in conjunction with his alcoholism. Theory based on the developmental psychopathology literature (see Cicchetti and Cohen 1995) indicates that family risk should be greatest when the parent’s psychopathological risk structure has been in place for most of his or her lifetime. On these grounds, and given the investigators’ interest in the role of parents’ antisocial behavior, a special variation (i.e., a developmental stipulation) was added: The distinction between subgroups had to be made not on the basis of an ASPD diagnosis but on the basis of the presence or absence of a sustained, high-level history of antisocial behavior during both childhood and adulthood. Men with a pattern of alcoholism in adulthood and a sustained lifetime history of high antisocial behavior were categorized as antisocial alcoholics (AAL’s). Those without such a sustained history were classified as non-antisocial alcoholics (NAAL’s).

Although this subtyping approach is similar to one based on an adult diagnosis of ASPD, it approaches the problem developmentally. It also takes into account a theory concerning the processes involved in the acquisition of alcohol abuse-dependence with this particular type of comorbidity pattern. A long history of research on the development of drinking problems has noted the occurrence of a variety of other forms of deviant behavior, including rule breaking, trouble making, and antisocial problem behavior, along with the drinking (Zucker et al. 1995*b*; Zucker et al. in press). In fact, this connection has been a central part of the dominant theories on the development of problem drinking behavior in adolescence (Kandel 1978). What is less well known is that for a subset of adolescents, this pattern begins substantially before adolescence and appears to continue into early adulthood and beyond. For another subset of youth, the pattern begins in adolescence but ends with the transition to adulthood, work roles, and marriage (see Zucker et al. 1995*b* for an extensive discussion of this literature).^5^

If it is effective, the AAL–NAAL subtyping strategy should reflect differences in the fathers’ lifetime AOD use (i.e., early and sustained involvement versus later onset and more transitory involvement). The AAL–NAAL subtypes also should serve as a marker for a variety of other influences that have shaped the early learning of the fathers’ alcohol-seeking and alcohol-using behavior. Thus, it would be expected that the AAL’s, more often than the NAAL’s, come from families with dense family histories of alcoholism and have been reared in environments that encouraged or caused them to seek the company of early AOD-using peers (Pihl and Peterson 1994; Johnson et al. 1995). If this typing strategy works, it also may prove useful as a marker of the different parenting activities of these men and their partners, which then may help to identify variations in their children’s risk for later alcohol problems.

The AAL–NAAL classification, based solely on the father’s alcoholism, was used to chart individual and familial characteristics pertaining to alcohol use and familial and social functioning. The classification strategy produced findings that largely were as predicted. Other derivative findings also emerged that supported the scheme’s validity. The analyses indicated that the scheme sorted out differences among the parents that likely will serve as markers of differing vulnerability for the children (see table 1). Moreover, the AAL’s and NAAL’s differed on several measures of the rearing environment that are apt to have an effect on the children’s socialization (see table 2). For example, the AAL men had denser family histories of alcoholism, lower levels of intellectual functioning,^6^ and significantly higher levels of nonalcoholic psychopathology than did the NAAL men (Bingham et al. 1996; Ellis 1994; Ichiyama et al. in press; Zucker et al. 1994; Zucker et al. in press). In addition, results provided evidence for the aggregation of risk by way of assortative mating^7^ among the AAL families. For example, the wives of AAL men had higher levels of antisocial behavior than did the wives of NAAL or control men. The AAL wives also had more nonantisocial psychopathology and higher lifetime levels of alcohol-related problems than did the wives of the control men, although they did not differ on these characteristics from the NAAL wives. Finally, the AAL parents displayed more aggressive behavior and conflict and were lower in socioeconomic status than were the NAAL and control families. Other analyses have shown that this is a result of downward social mobility rather than differences in social origin between the AAL’s and the NAAL’s (Zucker et al. in press).

Although the pattern of these findings is of considerable interest, concerns among scientists who have debated subtype issues focus on three vital questions. First, given that the two alcoholic types differ in level of antisocial behavior, what evidence exists that these differences are particular to the alcoholism? To answer this question one needs to determine if a sustained antisocial life-course subtype also exists among nonalcoholic populations. Current evidence indicates that this is highly unlikely, at least in this culture; because the link between antisocial behavior and alcoholism is so close, sustained antisocial behavior among nonalcoholics statistically is a rare occurrence (Zucker et al. in press).

Second, because the AAL’s and the NAAL’s differ in their levels of sustained antisocial behavior, is it more parsimonious to regard the high and low levels of antisocial behavior as extremes on a continuum, rather than as distinct types with similar (i.e., clustered) attributes? Several types of analysis, using sophisticated statistical techniques, have focused on this issue, including one analytic technique called configural cluster analysis (Zucker et al. in press) and another called structural equation modeling (Ellis 1994; Zucker et al. 1994), discussed below.

Results from the configural cluster analysis indicate that in addition to the close link between antisocial characteristics and alcoholism, one other distinct clustering, or type, is present. This type, called the nonantisocial alcoholic group, involves the coaggregation of alcoholism and a lifetime of continuous, low-level antisocial behavior. In other words, a pattern of continuous, high-level antisocial behavior is found in association with alcoholism; a pattern of continuous, low-level antisocial behavior is linked to the absence of alcoholism; and a third pattern, alcoholic coaggregation, also has been observed, in which low-level antisocial behavior is clustered with alcoholism.

Third, nonantisocial psychopathology as well as antisocial behavior varies across the two alcoholic subtypes. On these grounds, what evidence exists that the AAL–NAAL classification primarily involves higher versus lower levels of antisocial behavior, rather than variations in general psychopathology that occur over the life course? This is a central issue, because one of the major alcoholism subtyping schemes currently in use, the type A-type B categorization (Babor et al. 1992), is a framework that heavily categorizes alcoholism based on the level of psychopathology. To test this competing hypothesis, statistical analyses were conducted that removed the effects of general level/severity of psychopathology (Ichiyama et al. in press; Zucker et al. in press). The results still held, confirming the unique importance of the antisocial categorization.

## Outcomes Among the Children of Different Alcoholic Subtypes

The findings described in the previous section focus on parents in alcoholic families. These findings are consistent with other reports of differences between antisocial and nonantisocial forms of alcoholism in adults, and they expand on previous studies of family functioning. However, when considering factors that contribute to a child’s risk for later becoming alcoholic, one also needs to explore what impact the child’s functioning has within this family framework of risk. The proxy indicators being tracked by the MSU–UM study include measures of externalizing behavior problems (e.g., aggression and delinquent activity) as well as measures of internalizing behavior problems (e.g., schizoid/anxious, depressed, obsessive-compulsive, and uncommunicative behavior), hyperactivity (e.g., restlessness, short attention span, and fidgeting) and risky temperament (i.e., a composite index based on high activity level, emotional reactivity, and approach to life situations). To date, the study has collected data on the children and their families from two age periods: during preschool (i.e., ages 3 to 5) and during the early school years (i.e., ages 6 to 8).

Results show that during both early childhood and the early school years, significant behavioral differences exist between the children from families with different alcoholic subtypes (table 3) Bingham et al. 1996; Ellis 1994, Zucker et al. 1994). For example, externalizing behaviors, the foremost proxy indicator of the emergence of earlier and more problematic adolescent alcohol use, and internalizing behaviors are greatest among the children of AAL’s at both assessment periods. In addition, as preschoolers, the AAL boys showed more signs of hyperactivity and scored higher on a measure of risky temperament than did the boys from the NAAL and control families. Other analyses indicate that these differences exist not only in the level of overall group effects but in extremes of behavior. That is, significantly more boys from AAL homes than from the NAAL or control homes were classified in the clinical range on externalizing behavior problems (Jansen et al. 1995; Ellis 1994; Zucker et al. 1994). Finally, using a technique called structural equation modeling, researchers have found that separate process models for the AAL’s, NAAL’s, and control families better describe the interrelationships among the different variables than does one overall model. This finding implies that the pathways of influencing structure differ among the three groups and tentatively suggests that the mechanisms of risk development may be specific to subtypes. The latter finding has only been established at the first wave of data collection and will need to be replicated in later longitudinal analyses.

## The Broader Structure of Risk: Conclusions and Outlook

The typological classification described in this article and the derivative findings from the ongoing longitudinal study highlight observations from other investigators (e.g., Jacob and Leonard 1986), which indicate that not all alcoholic families are equally problematic and not all COA’s function in a manner that distinguishes them from nonalcoholic families or is indicative of a potentially troubled later outcome. For example, some elements of family functioning that are thought to be associated with alcoholism (e.g., aggression within the family) appear to be manifestations of only one subtype (i.e., AAL’s). Similarly, not all COA’s exhibit behaviors that differ from those of non-COA’s. Children from NAAL families occupy this intermediary position. From a practical standpoint, findings to date have indicated that NAAL families often are less identifiable as sources of developmental trouble, and the risk differences observed emphasize the possibility that NAAL children will be less at risk as they move into adolescence. The ability of researchers to determine more finely detailed and subtle differentiations within the alcoholic disorder is one aspect of the usefulness of subtyping.

The findings summarized here, which involved determining the families’ alcoholism subtypes as well as showing significant differences in childhood risk patterns related to the subtypes, were determined when the children were ages 3 to 5. It would be a serious mistake to conclude that all effects of subtyping and problematic outcome have appeared by the time these at-risk children have reached middle childhood. Evidence continues to indicate that both school and later peer influences play important roles in shaping a child’s risk status (Johnson et al. 1995); moreover, later positive or negative parental influences probably continue to sustain or alleviate child risk (Wills et al. 1996). Not all alcoholics remain actively alcoholic, and it is possible that the family subtype classifications used here will evolve over the course of childhood. Moreover, parents in alcoholic families frequently divorce, and new family structures may be formed that shape a child’s behavior in different ways from when he or she was young. This is a *probabilistic* framework for viewing how risk increases and decreases over time. It is important to keep this framework in mind, even as we discover that not all family structures carry the same risk burden.

At the same time, risk variation within subtypes is not random over time (Bingham et al, 1996), and the contextual structure that sustains and may even enhance an individual’s risk does not vary randomly either (Zucker et al. 1995*b*). Some social environments heavily restrict the range of opportunity; and, within these contexts, risk appears to be more heavily aggregated. Such restricted environments include poverty areas, frequently inhabited by disenfranchised minorities. The term “nesting environment” has been used to describe this restricted range of opportunity and the nonrandom aggregation of factors (i.e., nesting) that sustain individual risk. Under conditions of nestedness, when environment and biological risk coincide, subtypes are most likely to develop (also see Wills et al. 1996).

Two additional features of this research warrant some comment. First, not all aspects of early childhood functioning varied with familial subtype. For example, although preschool children from alcoholic families were more precocious in their ability to identify alcoholic beverages and exhibited a more highly developed conceptual understanding about alcohol as a drug, its effects, and who should use it, the subtype differences in the children’s development of these schemes were not evident (Zucker et al. 1995*a*). This finding was unexpected, given the earlier onset of drinking and drinking problems among adult AAL’s. It remains to be seen whether subtype differentiation will appear as the children grow older.

Second, the research carried out thus far has been guided by the proposition that one alcoholic subtype, marked by the sustained life-course presence of antisocial behavior, would differ from other forms of alcoholism in life-course functioning, in the rearing environment available to the offspring, and in childhood characteristics indicative of level of risk for later alcohol problems. Contrasts have been made against a heterogeneous group of other alcoholic families (NAAL’s), who in some respects are even indistinguishable from nonalcoholic families. Given the variety of other comorbid symptoms found among alcoholics in the general population, other subtypes may exist that display different distinguishing characteristics and which create different rearing and risk environments for their children. The comorbidity literature suggests that these characteristics may exist (Helzer et al. 1991; Zucker 1994), but other variants (e.g., alcoholism without comorbidity and developmentally limited alcoholism) have received much less attention in the typological literature. Accordingly, future research needs to better characterize these other variations among both fathers and mothers and to assess their influences on the development of risk among the children. Finally, researchers must determine the extent to which the effects of parental alcoholism subtype on the risk status of male COA’s can be generalized to female children from the same families.

## Figures and Tables

**Table 1:** Differences Among Families With Different Alcoholic Subtypes and Nonalcoholic Controls in Indicators of the Offspring’s Early Vulnerability for Alcoholism^1^

Indicators of Offspring’s Vulnerability	Degree to Which Indicator Is Present in Family
Family history of alcoholism	AAL > NAAL > Control
Paternal intellectual functioning	AAL < NAAL < Control
Maternal intellectual functioning	AAL < NAAL = Control

1 The indicators were measured when the children were preschool age (i.e., ages 3–5).

AAL = Antisocial alcoholics.

NAAL = Nonantisocial alcoholics.

Control = Matched nonalcoholics recruited from the same communities.

SOURCE: Adapted from Ellis et al. 1994 and Zucker et al. in press.

**Table 2:** Differences in Indicators of the Early Rearing Environment of Children From Families With Different Alcoholic Subtypes and From Nonalcoholic Control Families^1^

Indicator of Rearing Environment	Degree to Which Indicator Is Present in Family
Paternal Psychopathology	
Paternal current depression	AAL > NAAL = Control
Paternal worst-ever depression	AAL > NAAL > Control
Paternal lifetime alcohol problems	AAL > NAAL > Control
Maternal Psychopathology	
Maternal antisocial behavior	AAL > NAAL > Control
Maternal current depression	AAL > NAAL = Control
Maternal worst-ever depression	AAL = NAAL > Control
Maternal lifetime alcohol problems	AAL = NAAL > Control
Rearing Environment	
Family socioeconomic status	AAL < NAAL < Control
Maternal aggression toward spouse	AAL > NAAL = Control
Paternal aggression toward spouse	AAL > NAAL = Control

1 The indicators were measured when the children were preschool age (i.e., ages 3–5).

AAL = Antisocial alcoholics.

NAAL = Nonantisocial alcoholics.

Control = Matched nonalcoholics from the same communities.

SOURCE: Adapted from Ellis et al. 1994 and Zucker et al. in press.

**Table 3:** Differences in Childhood Risk Indicators Among Boys From Families With Different Alcoholic Subtypes and From Nonalcoholic Control Families

Childhood Risk Indicators	Degree to Which Indicator Is Present in Children
Preschool Years (ages 3–5)	
Child externalizing behavior problems^1^	AAL > NAAL > Control
Child internalizing behavior problems^2^	AAL > NAAL = Control
Child hyperactivity index^3^	AAL > NAAL = Control
Child risky temperament	AAL > NAAL = Control
Early School Years (ages 6–8)	
Child externalizing behavior problems	AAL > NAAL > Control
Child internalizing behavior problems	AAL > NAAL = Control

1 Externalizing behavior problems include aggressivity and delinquency.

2 Internalizing behavior problems include depressed or uncommunicative behavior.

3 The hyperactivity index measures characteristics such as restlessness and a short attention span.

AAL = Antisocial alcoholics.

NAAL = Nonantisocial alcoholics.

Control = Matched nonalcoholics from the same communities.

SOURCE: Adapted from Bingham et al. 1996 and Ellis et al. 1994.

## Footnotes

1 Throughout the remainder of this article, the term “alcoholism” is used to represent the clinical diagnosis of both alcohol abuse and alcohol dependence.

2 Because this alternative ultimately ties back to a common causal process for both disorders and thus would contribute to understanding subtypes and their outcomes, it can be considered a variant of the first alternative described.

3 In 1 percent of the families, a parent has died. When the study began, the researchers expected some attrition because of a lack of interest and an inability to locate families. However, the skill of the project’s clinically trained research staff and the regular contact through newsletters and birthday and Christmas cards have allowed the study to sustain this high level of family involvement.

4 The term “genetic vulnerability” specifically describes the measurement of particular genetic attributes.

5 The same two identifiably different life-course paths (i.e., trajectories) also have been noted in literature on the development of antisocial behavior (Moffitt 1993) and have given rise to a parallel taxonomy of subtypes with different onsets, life trajectories, and correlates.

6 The manner in which parental IQ differences contribute to a child’s risk is less obvious than for some of the other variables described here. However, lower IQ is related to the use of less reasoned and more authoritarian forms of child discipline and lesser parent agreement about child rearing and also may be related to specific differences in child-rearing practices, such as deficits in monitoring, that ultimately may contribute to the development of antisocial behavior in the child (Davies et al. 1989). Lower IQ also may be a low-level indicator of neural regulatory deficits that may be a part of the genetic predisposition that contributes to the non-alcohol-specific differences in impulsivity noted among COA’s (Martin et al. 1994).

7 Assortative mating is the nonrandom choice of a partner based on personal characteristics (e.g., an alcoholic is more likely than a nonalcoholic to have an alcoholic partner).
